# Ferroptosis related gene signature in T cell-mediated rejection after kidney transplantation

**DOI:** 10.1186/s12920-023-01440-y

**Published:** 2023-01-19

**Authors:** Weixun Zhang, Lian Gong, Di Zhang, Xiaopeng Hu

**Affiliations:** 1 Department of Urology, Beijing Chao-Yang Hospital, Capital Medical University, Beijing, China; 2 Institute of Urology, Capital Medical University, Beijing, China

**Keywords:** TCMR, Ferroptosis, Kidney transplantation, Allograft rejection, Medical bioinformatics

## Abstract

**Background:**

T cell-mediated rejection is an important factor affecting early transplant kidney survival. Ferroptosis has been shown to play a pathogenic role in a variety of diseases, which was not reported in TCMR. Here we developed a model for assessing activation of ferroptosis-related genes in TCMR to find a better screening method and explore the contribution of ferroptosis in TCMR.

**Methods:**

We performed unsupervised consensus clustering according to expression of ferroptosis-related genes based on RNA-seq data from kidney transplant biopsies, and developed an assessment model characterized by ferroptosis gene expression through PCA, which was evaluated in multiple external datasets as well as blood and urine samples. Pathway enrichment and immune cell infiltration analysis were used to explore the possible targets and pathways involved in ferroptosis and TCMR.

**Results:**

A ferroptosis gene expression scoring model was established. The diagnostic specificity and sensitivity of TCMR in renal biopsy samples were both over 80%, AUC = 0.843, and AUC was around 0.8 in multi-dataset validation, and was also close to 0.7 in blood and urine samples, while in predicting of graft survival at 3 years, scoring model had a good prognostic effect as well. Pathway enrichment and PPI network speculated that TLR4, CD44, IFNG, etc. may be the key genes of ferroptosis in TCMR.

**Conclusions:**

Ferroptosis scoring model could better diagnose TCMR and predict graft loss, and could be used as a potential screening method in blood and urine samples. We speculate that ferroptosis plays an important role in TCMR.

**Supplementary Information:**

The online version contains supplementary material available at 10.1186/s12920-023-01440-y.

## Background

Kidney transplantation is the optimal treatment for end-stage renal disease, it improved the quality of life and survival benefits significantly than dialysis. Benefiting from the development of immunosuppressants, the graft survival rate after kidney transplantation has been greatly improved [1]. However, deceleration of improvement in short-term graft survival appeared in last 20 years, 6.8% of adult kidney transplant recipients in 2018–2019 experienced acute rejection by 1 year [2]. T cell-mediated rejection (TCMR) plays an important role in the early stage of allograft rejection, despite a variety of immunosuppressive regimens targeting TCMR, the mechanisms underlying the occurrence and development of TCMR have not been well elucidated.

Ferroptosis is an iron-dependent form of regulated cell death caused by lipid peroxidation, which is controlled by integrated oxidation and antioxidant systems. It is responsible for numerous diseases, including bacterial infection, inflammatory bowel disease, tumorigenesis and therapy, ischemia reperfusion injury. CD8^+^ T cells, macrophages, IFN-γ and other key pathogenic factors in allograft rejection all have crosstalk with the ferroptosis process [3]. However, it remains unclear whether ferroptosis is involved and make pathogenic contribution in the process of renal allograft rejection.

In this study, we obtained six gene expression omnibus (GEO) datasets of renal transplantation, including biopsy, blood and urine samples. Through targeted analysis of ferroptosis-related genes, samples were classified according to the expression activity of ferroptosis genes, the classification ability of the new classification program was evaluated in multiple dimensions, aiming to establish a reliable TCMR diagnostic and prognostic model and clarify the potential key roles and intervention targets of ferroptosis in TCMR.

## Methods

### Patients and samples

**Figure1:**
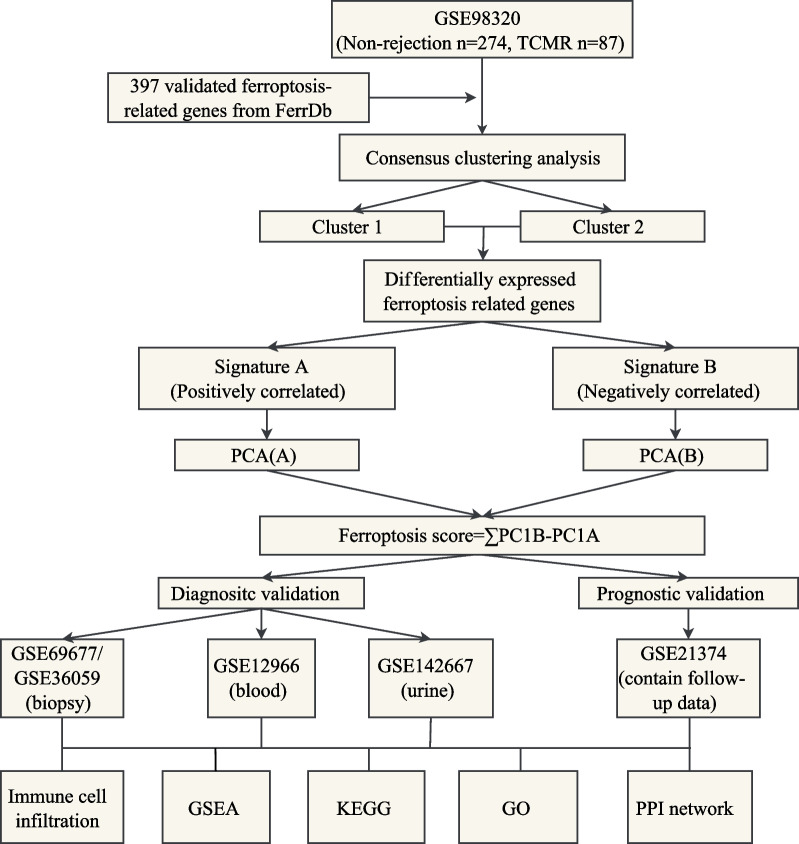
Flowchart of research design

A total of 1171 RNA sequencing data of kidney transplant patients were retrieved from GEO database (GSE98320, GSE69677, GSE129166, GSE142667, GSE36059 and GSE21374), biopsy, blood and urine samples were all included. Demographic characteristics of patients included were listed in Additional file 1: Table S5. GSE98320 was used for the establishment of the ferroptosis scoring model, and the rest datasets were for validation (Fig. 1, Additional file 1: Table S1). Data downloaded in this study is fully complied with the data access policies of GEO.

### Ferroptosis-based consensus clustering analysis

Consensus clustering analysis was based on 397 validated ferroptosis-related genes obtained from FerrDb (zhounan.org/ferrdb) on June 3^rd^ (Additional file 1: Table S2), and clustering process was accomplished by “ConsensusClusterPlus” R package.

### Generation of ferroptosis score

Differentially expressed genes (DEGs) among two groups clustered by consensus clustering analysis was screened by “limma” R package with adj. *p* < 0.05 and |logFC|> 0.5 (Additional file 1: Table S3). DEGs that are positively and negatively correlated with cluster classification were named signature A and signature B, respectively. Subsequently, based on “factoextra” R package, Principal Component Analysis (PCA) was performed for signature A and signature B. The definition of ferroptosis score is similar with Gene expression grade index [4], that is, the sum of principal component 1 (PC1) values of feature B minus the sum of PC1 values of feature A. (Ferroptosis score = ∑PC1B − ∑PC1A).

### External datasets validation

Datasets including acute TCMR (GSE69677, GSE36059), blood samples of TCMR(GSE129166) and urine samples of TCMR(GSE142667) were utilized for external validation. The ferroptosis score for each sample was calculated based on the rotation value in previous PCA analysis, and “ROCR” R package was applied for receiver operating characteristic curve (ROC) in each validation datasets, Area Under Curve (AUC) was calculated for evaluation. GSE21374 was obtained for survival predictive analysis based on “survival” R package.

### GSEA and immune infiltration

Samples were distinguished into two groups according to ferroptosis score, and Gene Set Enrichment Analysis (GSEA) was applied to elucidated the potential biological processes and pathways of those two groups with q-value < 0.05 and annotations of msigdb.v7.0.entrez.gmt.

Analysis of immune cell infiltration is based on CIBERSORT [5] (http://cibersort.stanford.edu/).

### GO and KEGG analysis

The screened DEGs between high and low ferroptosis score samples were analyzed by Gene Ontology (GO) enrichment and Kyoto Encyclopedia of Genes and Genomes (KEGG) enrichment [6–8], which were presented by the “clusterProfiler” R package.

### Construction of protein–protein interaction (PPI) network

All the protein–protein interactions with confidence score ≥ 0.7 in differentially expressed ferroptosis genes between high and low ferroptosis score samples were extracted from STRING database (https://cn.string-db.org/). Interaction relationships were clustered into two by k-means, proteins without interactions were deleted, and interaction line thickness indicates the strength of data support.

### Statistical analysis

Statistical analysis was conducted through R (version 4.2.1). Differences in immune cell infiltration levels and ferroptosis scores were assessed by student t-tests, *p* < 0.05 was considered statistically significant.

## Results

### Ferroptosis-related genes expression patterns in TCMR

**Fig. 2:**
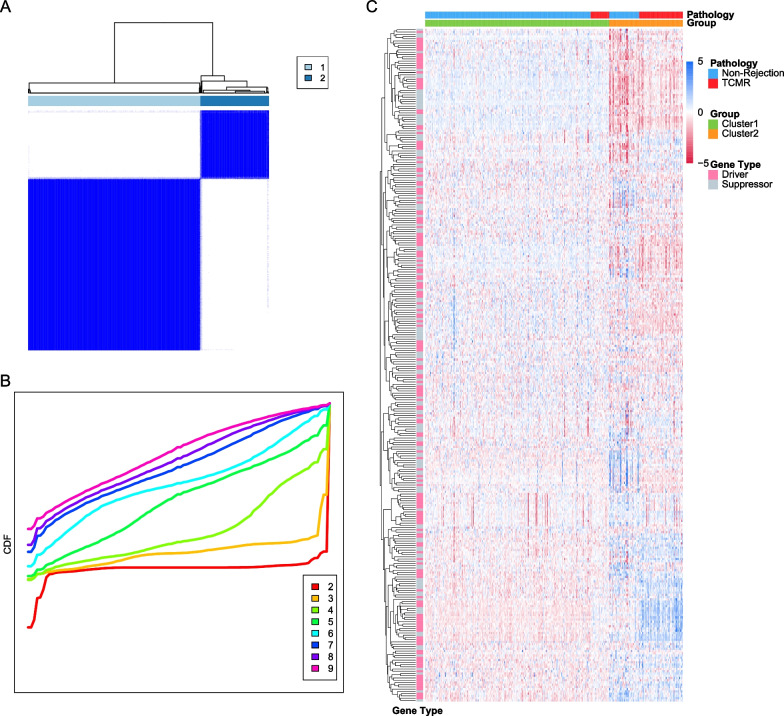
(**A**) Matrix heatmap of consensus clustering based on ferroptosis related genes. **B** Cumulative distribution curve of consensus clustering with k = 2–9. **C** Heatmap depicted the correlation between clusters and pathological features

**Fig. 3:**
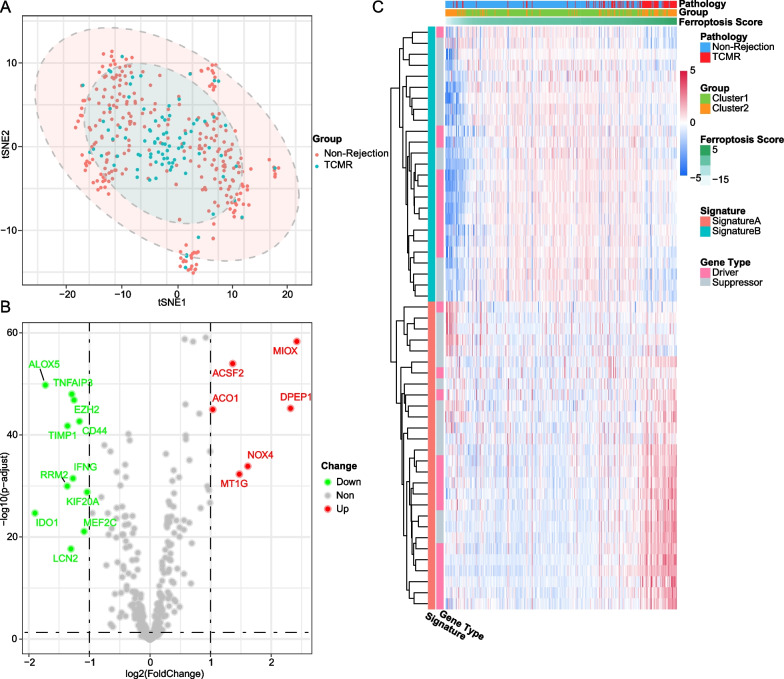
**A** TCMR and non-rejection samples cannot be well grouped on genome-wide level by tSNE plot. **B** DEGs of two clusters in ferroptosis-related genes shown by volcano plot. **C** Heatmap depicted the correlation between ferroptosis score and pathological features

Of dataset GSE98320, patients with non-rejection and TCMR confirmed by renal biopsy were selected, and 397 validated ferroptosis-related genes were retrieved in the ferroptosis database FerrDb, of which 198 were shown to be driver genes and 199 were shown to be suppressor genes. Unsupervised consistent clustering was performed based on ferroptosis-related genes. The cumulative distribution curve shows best clustering effect when the number of clusters is two, as the curve of k = 2 is most horizontal in the middle section (Fig. 2A, B). Heatmap shows the pathological types and gene expression levels corresponding to cluster 1 and cluster 2 (Fig. 2C), and majority of TCMR samples are clustered into cluster 2, as well as non-rejection samples in cluster 1. Both groups have different patterns in the expression of ferroptosis related driver genes and suppressor genes. We further performed T-distributed Stochastic Neighbor Embedding (tSNE) at the genome-wide level (Fig. 3A), as shown in the plot, TCMR and non-rejection samples were not well grouped, which means that TCMR do not have a clear degree of discrimination in the whole gene expression. However, ferroptosis-related gene expression clustering could better classify. To identify the leading DEGs of two clusters, we set |logFC|> 1 and adj. *p* value > 0.05 for accuracy. In cluster 2, which contains more TCMR samples, 6 genes were significantly up-expressed, of which 5 were ferroptosis driver genes, besides, 6 of the 11 down-expressed genes were ferroptosis suppressor genes, which infers that cluster 2 ferroptosis activity may be more inclined to the activated state (Fig. 3B). In short, there’s a separation trend in consistent clustering, but samples cannot be well differentiated perfectly based on clustering alone.

### Ferroptosis score have a better performance in differentiation of TCMR

**Fig. 4:**
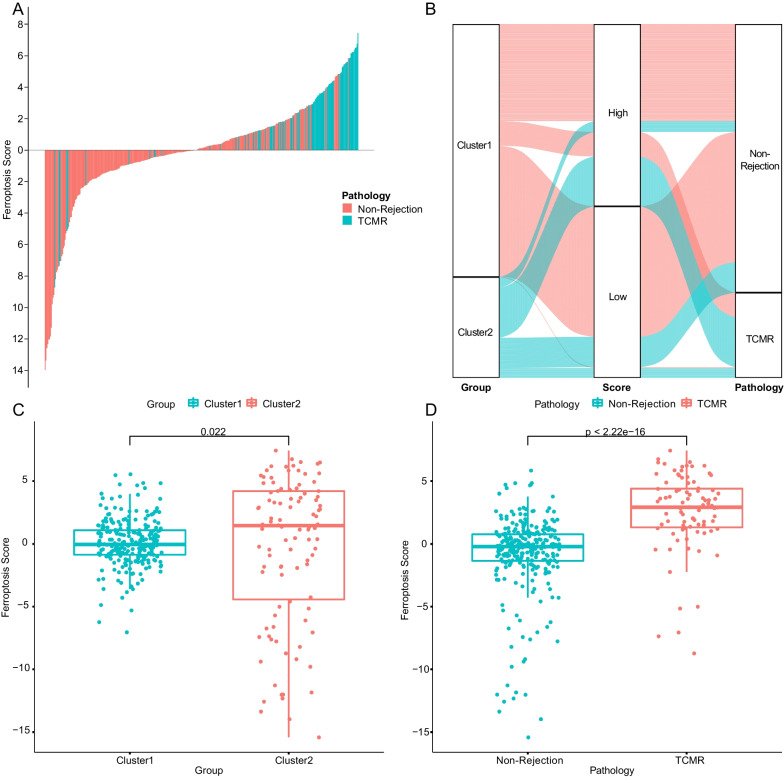
**A** TCMR samples concentrated on high ferroptosis score area. **B** Alluvial diagram of clusters, ferroptosis scores, and pathology features. **C**, **D** Ferroptosis scores are significantly higher in TCMR group as well as cluter2

To further increase the accuracy of the model, we expanded DEGs screening criterion to |logFC|> 0.5(Additional file 1: Table S3). The combination of up-expressed genes was named feature A, and the down-expressed genes were named feature B, PCA was performed on feature A and feature B respectively. Using a method similar to gene expression scoring index [4], the result of subtracting the PC1 values of Signature B and Signature A was used as ferroptosis score for represent the expression activity of ferroptosis signature genes (Additional file 1: Table S4). As shown in the heat map (Fig. 3C), after sorting the ferroptosis score from low to high, cluster 2 witch present higher ferroptosis activation is aggregated in extremely high or extremely low score area, and divided into two subgroups, most of TCMR samples gather in high-scoring area, and non-rejection samples in cluster2 mostly around low-scoring area. From the perspective of gene expression characteristics, the aforementioned non-rejection samples in cluster2 have significantly reduced gene expression levels in feature A than other samples, while TCMR samples in cluster2 were only slightly reduced. The difference in expression with the same trends but different levels may be the cause of inaccurate cluster analysis, however, it was corrected by our ferroptosis scoring model perfectly. Bar charts and boxplots provide a more visual representation of the association between ferroptosis scores and pathological outcomes (Fig. 4A–D).

### Validation of the ferroptosis score model

**Fig. 5:**
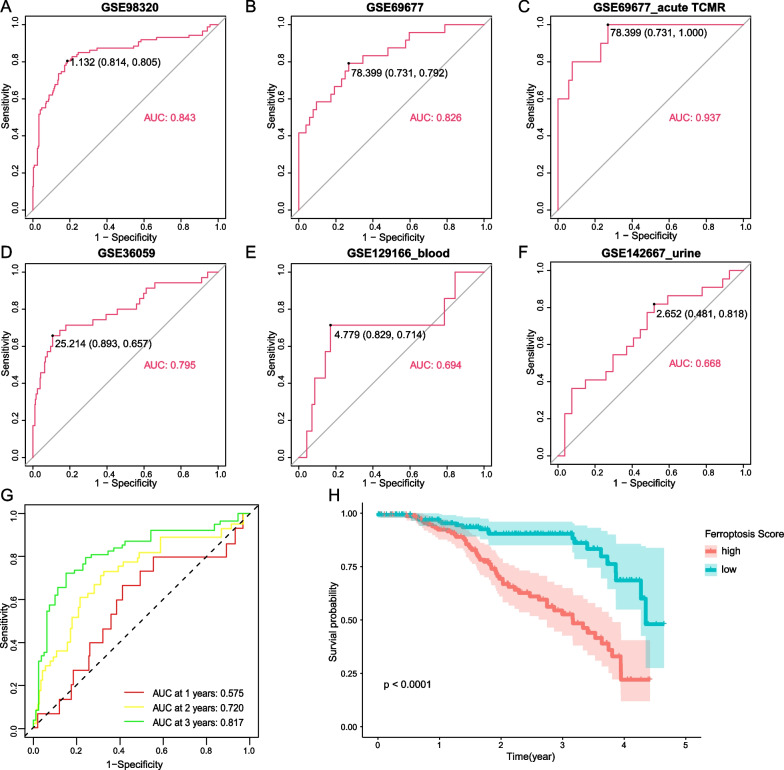
**A**–**F** Validation in biopsy samples (GSE98320, GSE69677, GSE36059), blood samples (GSE129166) and urine samples (GSE142667) by ROC. **G** ROC of kidney allograft survival. **H** Kaplan–Meier survival analysis for samples with high and low ferroptosis scores

In order to verify the generalizability of using ferroptosis score as a classification standard, we first tested ROC in the modeling dataset. Of GSE98320, the diagnostic specificity and sensitivity of the ferroptosis score for TCMR both over 80%, and AUC reached 0.843 (Fig. 5A). Subsequently, in other datasets, it was also proved that ferroptosis score has shown good diagnostic performance. In GSE69677 including acute/chronic TCMR and non-rejection samples, AUC could reach 0.826. Surprisingly, when acute TCMR is selected individually, ferroptosis scoring model showed better results with an AUC of 0.937 and a diagnostic sensitivity of 100%, which also means that ferroptosis-related pathological processes may be more active in acute TCMR (Fig. 5B, C). In another dataset containing 35 TCMR and 281 non-rejection biopsy samples, AUC reached close to 0.8 as well (Fig. 5D). Ferroptosis score was confirmed to have a satisfactory effect on the classification of TCMR in multiple datasets. Furthermore, we sought the diagnostic effect of ferroptosis scores in peripheral blood and urine samples (Fig. 5E, F). Of these two datasets, AUC were 0.694 and 0.668, respectively. Despite not as accurate as in biopsy samples, it could still be used as an alternative, and noninvasive screening method.

In addition to the diagnostic performance, we further assessed the predictive performance of the ferroptosis score for graft failure, and in the three-year survival rate, ferroptosis scoring model had the best predictive effect (Fig. 5G), and patients with low scores had better post-transplant survival (Fig. 5H).

### GSEA based on ferroptosis score

**Fig. 6:**
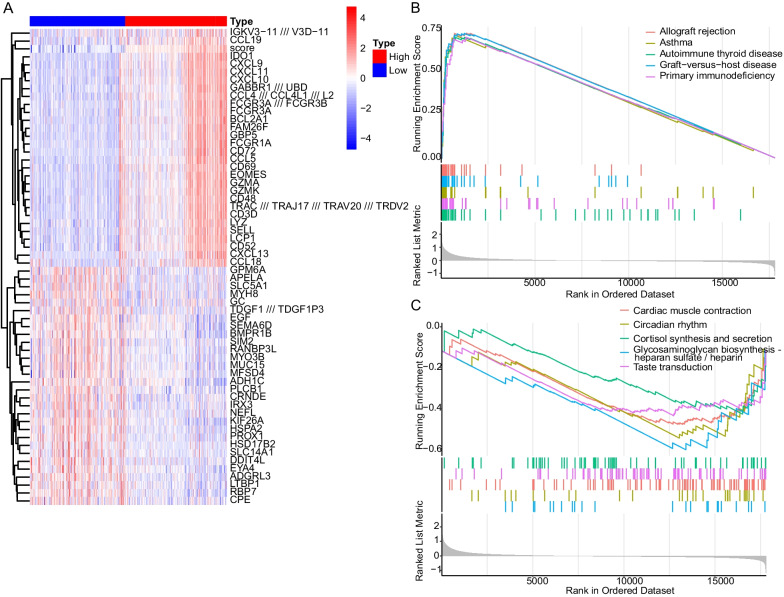
**A** Heatmap of DEGs between high and low score samples on genome-wide level with |logFC|> 1. **B** GSEA enriched pathways in high ferroptosis score group. **C** GSEA enriched pathways in low ferroptosis score group

We next screened DEGs between high and low score samples on genome-wide level, illustrates the distribution of DEGs after limiting |logFC| to 1 or more (Fig. 6A), and most of DEGs were immune chemotaxis-related genes. Subsequently, GSEA was performed on genome-wide expression of the samples with ferroptosis score used as the basis for grouping. Transplant rejection, asthma, autoimmune thyroid disease, graft-versus-host disease and Primary immunodeficiency were significantly enriched in the high-scoring samples. Of low-scoring samples, it may be attributed to poor sample heterogeneity, resulting in poor pathway enrichment effect, enriched in 5 pathways including cardiac muscle contraction, circadian rhythm, cortisol synthesis and secretion, glycosaminoglycan biosynthesis and taste transduction (Fig. 6B, C).

### The ferroptosis score-related landscape of immune cell infiltration

**Fig. 7:**
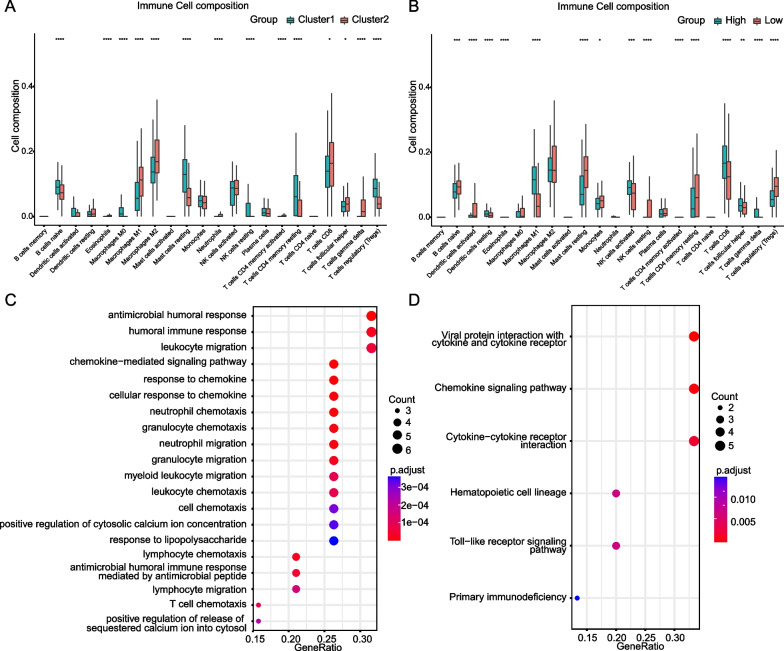
**A**, **B** Analysis of immune cell infiltration in clusters and ferroptosis score groups. **C** GO enrichment analysis and **D** KEGG enrichment analysis of DEGs

In CIBERSORT-based immune cell infiltration analysis, the levels of macrophages, memory T cells, Treg cells and other cells were significantly different when grouped by clusters, cluster2 showed stronger T cell immune activation status, with more similar immune cell infiltration patterns to TCMR (Fig. 7A). Of analysis grouped by ferroptosis score, the samples in the high score group also showed a strong T cell-related immune response, the number of M1 macrophages was significantly reduced, with a higher level of CD8^+^ T cells. In terms of the characteristics of immune cell distribution, high score group is more similar to the TCMR process (Fig. 7B).

### Pathway enrichment and protein–protein interaction for ferroptosis-related patterns

**Fig. 8:**
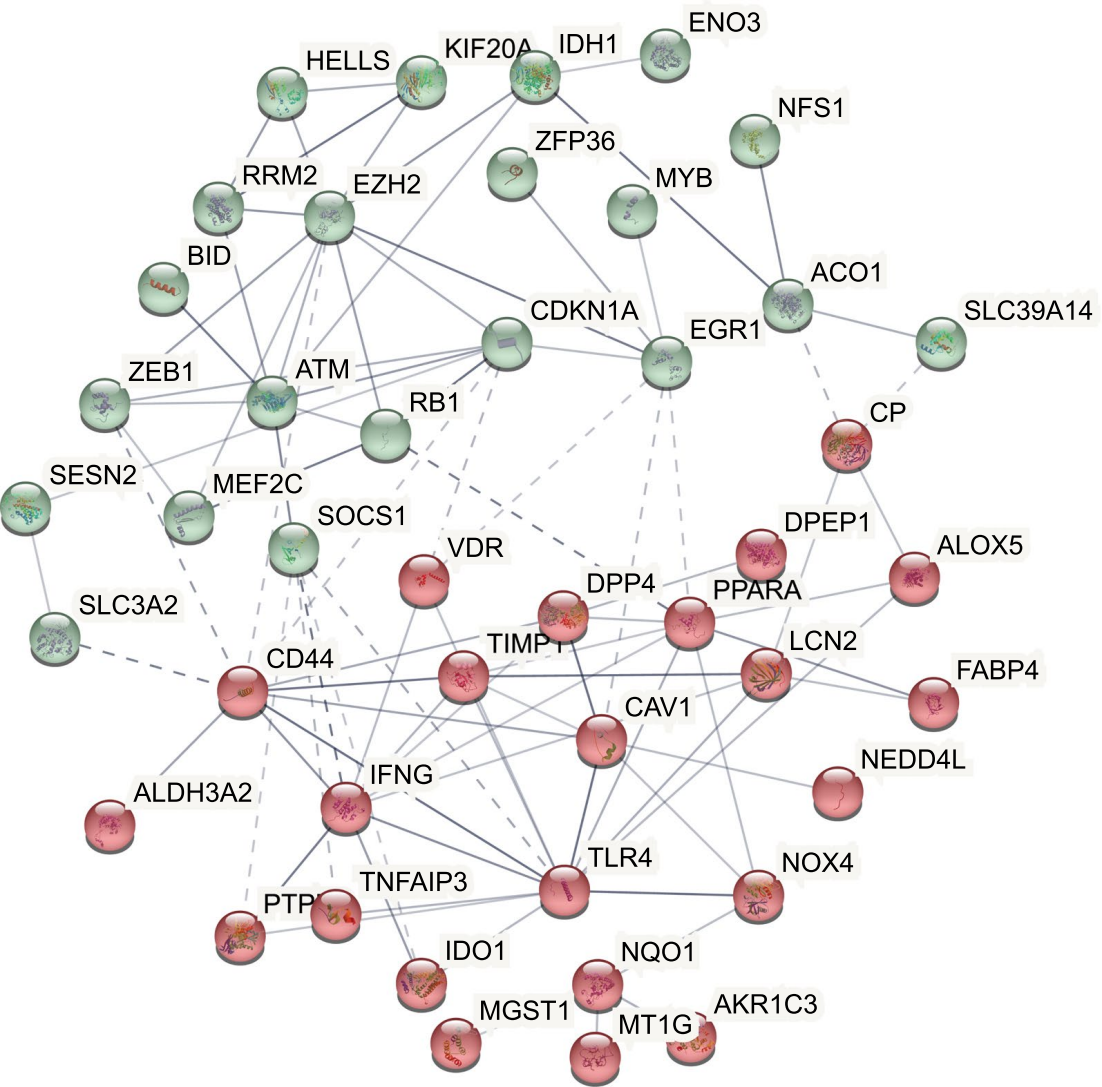
Protein–protein interactions network of differentially expressed ferroptosis genes between high and low score samples

Finally, in order to screen potential intervention pathways associated with ferroptosis-related genes in TCMR treatment, we performed GO, KEGG and PPI network analysis. The significant GO functional terms were mostly associated with the response to anti-infection, immune cell migration, such as antimicrobial humoral response, leukocyte migration and chemokine-mediated signaling pathway (Fig. 7C). KEGG analysis showed that it was also enriched in infection-related cytokines, chemokine-related pathways, and infection-related Toll-like receptor pathways (Fig. 7D). Of PPI network analysis, TLR4, CD44, EZH2, IFG and CDKN1A were shown as potential hub genes (Fig. 8).

## Discussion

TCMR is still one of the most important factors limiting the survival rate of kidney transplantation. Early diagnosis and interventions are effective ways to improve the survival rate of kidney allograft. However, due to the dominance of pathological biopsy in TCMR diagnosis, there is a lack of simple and effective screening and diagnosis methods. Ferroptosis has been proven to be associated with a variety of diseases, such as ischemia–reperfusion injury, tumors, acute kidney injury, etc.[9–11]. Our study preliminarily confirmed the reliable diagnostic and prognostic effect of ferroptosis-related gene expression scores in TCMR, and provided a new intervention target for TCMR.

Utilizing RNA-seq datasets of TCMR and non-rejection samples, combined with gene expression rank scoring through unsupervised consensus clustering, we constructed a scoring model that could reliably discriminate TCMR, which was validated in multiple TCMR kidney biopsy datasets, as well as blood and urine datasets. On the other hand, the ferroptosis score also showed a good predictive effect in respect of graft survival time.

To date, although ferroptosis has been extensively studied in various kidney diseases, there are still few studies on ferroptosis in kidney allograft loss [12], and none in TCMR. In addition to diagnostic and prognostic value, our model also provided preliminary speculation on the pathological mechanism of TCMR. The changes in the expression activity level of ferroptosis-related genes that we demonstrated were specifically altered in TCMR samples, implying that in the process of TCMR, ferroptosis may be relatively activated or inhibited compared to non-rejection samples. Ferroptosis may occur in primarily damaged cells such as tubular epithelial cells, or immune cells such as CD8^+^ T cells, or other types of cell populations, which in turn play different disease-promoting or inhibitory roles [13, 14].

From our enrichment analysis of differential expressed ferroptosis genes between high and low score groups, key pathways were concentrated on infection response and immune cell chemotaxis and migration-related pathways. Combining with the results of protein–protein interaction network, TLR4 may act as an important intermediate link of ferroptosis process involved in TCMR.

Toll-like receptor 4 (TLR4) is a pattern recognition receptor of innate immunity that recognizes and defends against invading microorganisms. TLR4 is expressed on a variety of cells in kidney, including immune cells such as macrophages, T and B lymphocytes, and non-immune cells such as renal tubular epithelial cells and endothelial cells [14]. Interactions of TLR4 and T cells exist in various diseases. In tumors, C–C motif chemokine receptor-like 2 (CCRL2) enhances TLR4-mediated inflammatory signaling by interacting with membrane TLR4, thereby enhancing anti-tumor T cell responses [15]. A steady-state suboptimal TLR4 response may protect the transplant recipient from rejection by minimizing the signaling for dendritic cell maturation and subsequent T-cell activation [16]. The relationship of ferroptosis and transplantation have also been elucidated by recent studies. In cardiac transplantation, ferroptosis promotes neutrophil recruitment after cardiac transplantation through the TLR4/Trif/IFN signaling pathway [17]. To sum up, several studies have shown the existence of crosstalk between TLR4, ferroptosis and immune cells, and our analysis has drawn similar conclusions, but it is still unknown the exact role ferroptosis plays in TCMR.

Another target screened by enrichment analysis, CD44, is another cell-surface adhesion molecule that activates T cell responses following transplantation. Inhibition of CD44 with mAbs can suppress accelerated heart allograft rejection in mice [18, 19], and down-regulation of CD44 could inhibit acute cellular rejection in kidney allograft [20], which can also be used as a potential intervention target.

In summary, the ferroptosis signature genes we identified have a high value in diagnosis of TCMR occurrence after kidney transplantation and prediction of graft loss, but the mechanisms of ferroptosis process in TCMR remain to be further investigated.

## Conclusions

In conclusion, we have established a scoring model based on the activation of ferroptosis-related genes, which can better diagnose TCMR and predict graft loss, and could be used as a potential screening method in blood and urine samples. Our study also initially clarified the possible important role of ferroptosis in acute cell-mediated rejection, but its mechanism still needs more in-depth exploration.

## Supplementary Information

**Additional file 1: Table S1.** Details of datasets. **Table S2.** Ferroptosis related genes list. **Table S3.** DEGs in cluster1 and cluster2. **Table S4.** Rotation of PCA. **Table S5.** Demographic characteristics

## Data Availability

The data that support the findings of this study are available in additional file, RNA-seq datasets from gene expression omnibus are available on https://www.ncbi.nlm.nih.gov/geo/ (GSE98320, https://www.ncbi.nlm.nih.gov/geo/query/acc.cgi?acc=GSE98320; GSE69677, https://www.ncbi.nlm.nih.gov/geo/query/acc.cgi?acc=GSE69677; GSE129166, https://www.ncbi.nlm.nih.gov/geo/query/acc.cgi?acc=GSE129166; GSE142667, https://www.ncbi.nlm.nih.gov/geo/query/acc.cgi?acc=GSE142667; GSE36059, https://www.ncbi.nlm.nih.gov/geo/query/acc.cgi?acc=GSE36059; GSE21374, https://www.ncbi.nlm.nih.gov/geo/query/acc.cgi?acc=GSE21374), further inquiries can be directed to the corresponding author.

## Abbreviations

TCMR: T cell-mediated rejection
GEO: Gene Expression Omnibus
DEGs: Differentially Expressed Genes
PCA: Principal Component Analysis
PC1: Principal Component 1
ROC: Operating Characteristic Curve
AUC: Area Under Curve
GSEA: Gene Set Enrichment Analysis
GO: Gene Ontology
KEGG: Kyoto Encyclopedia of Genes and Genomes
PPI: Protein–protein interaction
tSNE: T-distributed Stochastic Neighbor Embedding
TLR4: Toll-like receptor 4
